# Application of interpretable machine learning algorithms to predict distant metastasis in ovarian clear cell carcinoma

**DOI:** 10.1002/cam4.7161

**Published:** 2024-04-12

**Authors:** Qin‐Hua Guo, Feng‐Chun Xie, Fang‐Min Zhong, Wen Wen, Xue‐Ru Zhang, Xia‐Jing Yu, Xin‐Lu Wang, Bo Huang, Li‐Ping Li, Xiao‐Zhong Wang

**Affiliations:** ^1^ Jiangxi Province Key Laboratory of Laboratory Medicine, Jiangxi Provincial Clinical Research Center for Laboratory Medicine, Department of Clinical Laboratory, The Second Affiliated Hospital Jiangxi Medical College, Nanchang University Nanchang Jiangxi China; ^2^ Department of Clinical Laboratory The First Hospital of Nanchang (The Third Affiliated Hospital of Nanchang University) Nanchang Jiangxi China; ^3^ School of Public Health Nanchang University Nanchang Jiangxi China; ^4^ Department of Clinical Laboratory Nanchang Renai Obstetrics and Gynecology Hospital Nanchang Jiangxi China

**Keywords:** machine learning, metastasis, ovarian clear cell carcinoma, SEER

## Abstract

**Background:**

Ovarian clear cell carcinoma (OCCC) represents a subtype of ovarian epithelial carcinoma (OEC) known for its limited responsiveness to chemotherapy, and the onset of distant metastasis significantly impacts patient prognoses. This study aimed to identify potential risk factors contributing to the occurrence of distant metastasis in OCCC.

**Methods:**

Utilizing the Surveillance, Epidemiology, and End Results (SEER) database, we identified patients diagnosed with OCCC between 2004 and 2015. The most influential factors were selected through the application of Gaussian Naive Bayes (GNB) and Adaboost machine learning algorithms, employing a Venn test for further refinement. Subsequently, six machine learning (ML) techniques, namely XGBoost, LightGBM, Random Forest (RF), Adaptive Boosting (Adaboost), Support Vector Machine (SVM), and Multilayer Perceptron (MLP), were employed to construct predictive models for distant metastasis. Shapley Additive Interpretation (SHAP) analysis facilitated a visual interpretation for individual patient. Model validity was assessed using accuracy, sensitivity, specificity, positive predictive value, negative predictive value, F1 score, and the area under the receiver operating characteristic curve (AUC).

**Results:**

In the realm of predicting distant metastasis, the Random Forest (RF) model outperformed the other five machine learning algorithms. The RF model demonstrated accuracy, sensitivity, specificity, positive predictive value, negative predictive value, F1 score, and AUC (95% CI) values of 0.792 (0.762–0.823), 0.904 (0.835–0.973), 0.759 (0.731–0.787), 0.221 (0.186–0.256), 0.974 (0.967–0.982), 0.353 (0.306–0.399), and 0.834 (0.696–0.967), respectively, surpassing the performance of other models. Additionally, the calibration curve's Brier Score (95%) for the RF model reached the minimum value of 0.06256 (0.05753–0.06759). SHAP analysis provided independent explanations, reaffirming the critical clinical factors associated with the risk of metastasis in OCCC patients.

**Conclusions:**

This study successfully established a precise predictive model for OCCC patient metastasis using machine learning techniques, offering valuable support to clinicians in making informed clinical decisions.

## 1 INTRODUCTION

Epithelial ovarian cancer (EOC), often referred to as ovarian cancer, comprises a heterogeneous group of diseases characterized by distinct genomic features.^1^ This malignancy exhibits a proclivity for chemotherapy resistance, a high recurrence rate, and a dismal prognosis, making it a leading cause of mortality among gynecological cancers.^1^, ^2^, ^3^ Based on their unique pathological characteristics, epithelial ovarian cancers can be classified into several subtypes, including ovarian serous carcinoma (OSC), ovarian mucinous carcinoma (OMC), ovarian endometrioid carcinoma (OEC), and ovarian clear cell carcinoma (OCCC).^3^, ^4^ OCCC is distinguished by the presence of transparent cytoplasm.^5^, ^6^ OCCC accounts for approximately 5%–20% of all ovarian cancer cases, with notable variations in incidence across different racial and geographical groups. Its prevalence is approximately 3.1% in the black population, 4.8% in the white population, and 11.1% among individuals of Asian descent.^7^, ^8^ Notably, the Asian region, particularly Japan, exhibits the highest incidence of OCCC, with rates reaching up to 25%.^9^

While OCCC is relatively straightforward to diagnose at an early stage, its inherent insensitivity to platinum‐based chemotherapy regimens contributes to a heightened risk of infiltration, metastasis, and relapse after treatment, resulting in a markedly unfavorable prognosis.^10^ Treatment modalities for OCCC recurrence and metastasis include secondary tumor cell reduction (SCS) and nonplatinum single‐agent chemotherapy, sometimes in combination with bevacizumab.^11^, ^12^ Clinical data have indicated that there is no substantial disparity in the 5‐year overall survival rate between 144 patients with recurrent metastatic OCCC who did not undergo SCS and 25 patients who did.^13^ Another study reported a notably low response rate of just 33% among 25 patients with platinum‐resistant OCCC who received secondary nonplatinum chemotherapy.^14^ Hence, the identification of risk factors for OCCC metastasis and recurrence, along with the development of a metastasis prediction model, is of paramount importance in improving the survival prospects of OCCC patients.

This research aims to extract clinical characteristics and treatment data of OCCC patients diagnosed between 2004 and 2015 from the SEER database. The study endeavors to analyze the factors influencing distant metastasis in OCCC patients and assess the prognostic indicators for patients with distant metastasis. Additionally, the study will construct nomograms to predict the risk and prognosis of distant metastasis in OCCC cases, providing valuable insights for guiding the clinical management and individual survival prognosis of patients with distant metastasis of OCCC.

## 2 METHODS

### 2.1 Database

**FIGURE 1:**
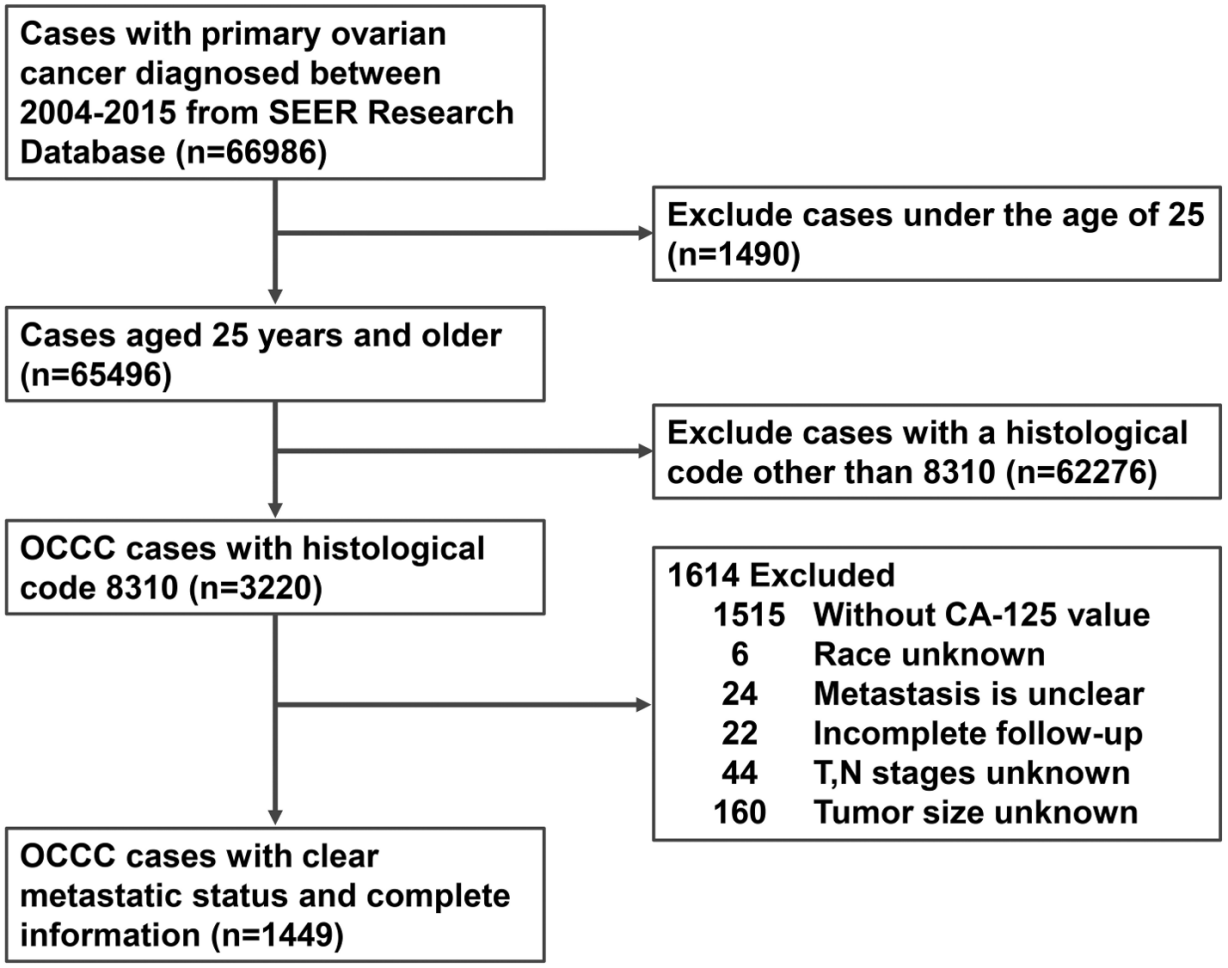
Data extraction flowchart from the SEER database.

The patient data utilized in this study was sourced from the SEER database (http://seer.cancer.gov/), SEER database is one of the authoritative large‐scale tumor registration databases in the United States. We retrievaled the clinical case data for OCCC patients aged 25 years or older from 2004 to 2015 from SEER database by SEER*Stat software (v 8.4.2), and export the data list to Excel table for the purposes of data screening (Figure 1).

### 2.2 Study population

We conducted a comprehensive data collection process spanning the years 2004–2015, involving 66,986 individuals diagnosed with ovarian cancer, as delineated by the International Classification of Diseases Version 10 (ICD‐10) code in the SEER database, signifying a confirmed diagnosis of ovarian cancer. To establish a well‐defined and homogeneous cohort for subsequent analysis, we established specific inclusion and exclusion criteria. The inclusion criteria necessitated those individuals be at least 25 years of age, possess ovarian clear cell carcinoma (OCCC) as their primary tumor, maintain a comprehensive record of essential personal and disease‐related information, exhibit clarity in terms of metastatic status, and have their diagnosis confirmed through pathological examination utilizing the ICD‐O‐3 HIST/BEHAV code “8310”. Conversely, exclusion criteria encompassed patients below 25 years of age, those lacking accurate metastatic information, individuals with a primary tumor other than OCCC, those without complete information records, and patients whose diagnosis relied on autopsy or a death certificate. Data extraction flowchart is shown as Figure 1.

This meticulous selection process resulted in a final cohort of 66,986 ovarian cancer patients, of which 3220 individuals had OCCC as their primary tumor. Among these, 1449 cases have metastases information. Our analysis incorporated demographic variables such as age, marital status, and race, in addition to clinicopathological and treatment‐related parameters, including tumor laterality, tumor size, preoperative serum CA125 levels, tumor differentiation (grades I, II, III, and IV, respectively, represent the degree of tumor differentiation as well differentiated, moderately differentiated, poorly differentiated, and undifferentiated. we define levels I and II as a group, and levels III and IV as a group), TNM stage, residual lesion size, surgical procedures, radiotherapy, and chemotherapy.

### 2.3 Weighting and selection of variables for analysis

Variable selection, namely the ranking of variable importance (weights), plays a crucial role in various predictive problems, encompassing dataset analysis and variable selection processes. Traditional methods of importance ranking primarily fall into two main categories: those based on model coefficients and those based on model performance (e.g., importance analysis using univariate numerical permutation).^15^ This study adopts the first category, which relies on model coefficients, to analyze variable importance. It employs machine learning techniques to rank the variables after the initial screening, thereby further extracting significant features. Three machine learning methods, namely GNB and Adaboost, are used for feature importance ranking.

### 2.4 Description of multiple machine learning algorithms

XGBoost is a supervised learning algorithm that combines weak regression trees to enhance predictive performance while controlling model complexity, utilizing a Taylor expansion focusing on the first and second derivatives of the error function. Applied in medicine, XGBoost addresses overfitting and reduces computational load.^16^ LightGBM, a Microsoft‐provided algorithm, is an iterative boosting tree system that improves upon traditional gradient boosting decision trees by utilizing both first‐ and second‐order negative gradients. It incorporates a Histogram‐based decision tree algorithm for faster execution and adopts a leaf‐wise growth strategy for increased efficiency.^17^ Random Forest (RF) is an ensemble learning algorithm that constructs a classification model using multiple decision trees. It employs a majority rule voting mechanism, providing high accuracy, resistance to interference, and ease of implementation but comes with a higher computational burden.^18^ Adaboost is an iterative algorithm that boosts weak classifiers by updating the weights of training samples, emphasizing misclassified samples in each iteration. It creates multiple weak classifiers, which are combined into a strong classifier through weighted summation.^19^ Support Vector Machine (SVM) is a classification algorithm minimizing structured risk for improved generalization, acting as a binary classifier and defining a maximum‐margin linear classifier in the feature space. SVM transforms into solving a convex quadratic programming problem, ensuring robust statistical patterns even with limited sample sizes.^20^ Multilayer Perceptron (MLP) addresses linearly inseparable problems by stacking multiple layers of linear classifiers with nonlinear activation functions. It includes input, hidden, and output layers characterized by weights, biases, and activation functions, commonly employing the Sigmoid function for nonlinear mappings within the specified output range.^21^

### 2.5 Statistical analysis and model evaluation

Concerning baseline characteristics, we utilized Student's *t*‐test or Mann–Whitney U test to compare quantitative data, while Fisher's exact test or chi‐squared test was employed for comparing qualitative data. The top 10 most critical risk factors for distant metastasis of ovarian cancer were identified through the application of GNB and Adaboost, and their eight intersection points were selected through Venn diagrams. Additionally, six machine learning classification algorithms, including XGBoost, LGBM, RF, AdaBoost, SVM, and MLP, were employed to establish a classifier model for distant metastasis. The dataset was divided into two parts: data from 2010 to 2014 were used for training, while data from 2015 were used for testing. The training dataset was utilized for selecting the optimal model, and the testing dataset was employed for model test. Model performance was assessed using metrics such as accuracy, sensitivity, specificity, positive predictive value, negative predictive value, F1 score, and the area under the receiver operating characteristic curve (AUC). Spearman correlation analysis is used to analyze the correlation between variables (+1 means a perfect positive association, −1 means a perfect negative association, If the absolute value of the correlation coefficient is less than 0.5, it indicates weak correlation). All statistical analyses were conducted using R version 3.6.3 and Python version 3.7, and results were deemed statistically significant at a threshold of *p* < 0.05 for all analyses.

## 3 RESULTS

### 3.1 Basic characteristics of training and test set

A total of 1449 OCCC cases were included. Data from 1170 patients between 2010 and 2014 were allocated to the training set, while data from 279 patients in 2015 were designated as the test set. Within the training set, distant metastases in OCCC patients accounted for 7.094% (83 cases), whereas in the test set, the proportion was 4.659% (13 cases). The basic characteristics of the data, classified into training and test set, are summarized in Table 1.

**TABLE 1:** Demographic and clinicopathologic variables within the entire cohort stratified based on training and test sets.

Variable	Total (*n* = 1449)	Training set (*n* = 1170)	Test set (*n* = 279)	*p*
Age, median [IQR]	57.0 [50.0, 63.0]	57.0 [49.0, 63.0]	57.0 [50.0, 64.0]	0.441
Race, *n* (%)				
Black	65 (4.486)	51 (4.359)	14 (5.018)	0.699
White	1067 (73.637)	867 (74.103)	200 (71.685)	
Other	317 (21.877)	252 (21.538)	65 (23.297)	
Marital, *n* (%)				
Yes	767 (52.933)	621 (53.077)	146 (52.330)	0.061
No	374 (25.811)	289 (24.701)	85 (30.466)	
Unknown	308 (21.256)	260 (22.222)	48 (17.204)	
Laterality, *n* (%)				
Two‐sided	164 (11.318)	137 (11.709)	27 (9.677)	0.437
One‐sided	1271 (87.716)	1023 (87.436)	248 (88.889)	
Paired	14 (0.966)	10 (0.855)	4 (1.434)	
Tumor size, median [IQR]	114.0 [70.0,160.0]	110.0 [70.0,160.0]	120.0 [72.00, 150.0]	0.721
CA125, *n* (%)				
Normal	312 (21.532)	244 (20.855)	68 (24.373)	0.301
Upper	785 (54.175)	634 (54.188)	151 (54.122)	
Unknown	352 (24.293)	292 (24.957)	60 (21.505)	
Residual Tumor Volume, *n* (%)				
Without	857 (59.144)	682 (58.291)	175 (62.724)	0.263
≤1 cm	51 (3.520)	43 (3.675)	8 (2.867)	
>1 cm	35 (2.415)	32 (2.735)	3 (1.075)	
Unknown	506 (34.921)	413 (35.299)	93 (33.333)	
Grade, *n* (%)				
I‐II	120 (8.282)	97 (8.291)	23 (8.244)	0.870
III‐IV	932 (64.320)	749 (64.017)	183 (65.591)	
Unknown	397 (27.398)	324 (27.692)	73 (26.165)	
T, *n* (%)				
T1	964 (66.529)	776 (66.325)	188 (67.384)	0.819
T2	201 (13.872)	161 (13.761)	40 (14.337)	
T3	284 (19.600)	233 (19.915)	51 (18.280)	
N, *n* (%)				
N0	1271 (87.716)	1023 (87.436)	248 (88.889)	0.506
N1	178 (12.284)	147 (12.564)	31 (11.111)	
M, *n* (%)				
M0	1353 (93.375)	1087 (92.906)	266 (95.341)	0.142
M1	96 (6.625)	83 (7.094)	13 (4.659)	
Radiation, *n* (%)				
No	1426 (98.413)	1152 (98.462)	274 (98.208)	0.761
Yes	23 (1.587)	18 (1.538)	5 (1.792)	
Chemotherapy, *n* (%)				
No	277 (19.117)	230 (19.658)	47 (16.846)	0.283
Yes	1172 (80.883)	940 (80.342)	232 (83.154)	
Surgery, *n* (%)				
No	13 (0.897)	9 (0.769)	4 (1.434)	0.290
Yes	1436 (99.103)	1161 (99.231)	275 (98.566)	

*Note*: M1: represents a patient with distant metastasis.

### 3.2 The basic characteristics of metastatic and nonmetastatic data

Among a total of 1449 cases, 96 exhibited distant metastasis, while 1353 cases showed no signs of distant metastasis. Table 2 presents a comprehensive demographic and clinical analysis based on the criterion of distant metastasis.

**TABLE 2:** Demographic and clinicopathologic variables within the entire cohort stratified based on metastasis status.

Variable	Total (*n* = 1449)	Distant metastasis (−) (*n* = 1353)	Distant metastasis (+) (*n* = 96)	*p*
Age, median [IQR]	57.0 [50.0, 63.0]	56.000 [49.0, 63.0]	59.0 [51.0, 66.0]	0.165
Race, *n* (%)				
Black	65 (4.486)	60 (4.435)	5 (5.208)	0.530
White	1067 (73.637)	1001 (73.984)	66 (68.750)	
Other	317 (21.877)	292 (21.582)	25 (26.042)	
Marital, *n* (%)				
Yes	767 (52.933)	724 (53.511)	43 (44.792)	0.255
No	374 (25.811)	345 (25.499)	29 (30.208)	
Unknown	308 (21.256)	284 (20.990)	24 (25.000)	
Laterality, *n* (%)				
Two‐sided	164 (11.318)	134 (9.904)	30 (31.250)	<0.001
One‐sided	1271 (87.716)	1209 (89.357)	62 (64.583)	
Paired	14 (0.966)	10 (0.739)	4 (4.167)	
Tumor size, median [IQR]	114.0 [70.0, 160.000]	114.0 [70.0, 160.0]	114.0 [60.0, 150.0]	0.578
CA125, *n* (%)				
Normal	312 (21.532)	305 (22.542)	7 (7.292)	<0.001
Upper	785 (54.175)	716 (52.919)	69 (71.875)	
Unknown	352 (24.293)	332 (24.538)	20 (20.833)	
Residual Tumor Volume, *n* (%)				
Without	857 (59.144)	823 (60.828)	34 (35.417)	<0.001
≤1 cm	51 (3.520)	38 (2.809)	13 (13.542)	
>1 cm	35 (2.415)	25 (1.848)	10 (10.417)	
Unknown	506 (34.921)	467 (34.516)	39 (40.625)	
Grade, *n* (%)				
I‐II	120 (8.282)	114 (8.426)	6 (6.250)	0.112
III‐IV	932 (64.320)	877 (64.819)	55 (57.292)	
Unknown	397 (27.398)	362 (26.755)	35 (36.458)	
T, *n* (%)				
T1	964 (66.529)	948 (70.067)	16 (16.667)	<0.001
T2	201 (13.872)	185 (13.673)	16 (16.667)	
T3	284 (19.600)	220 (16.260)	64 (66.667)	
N, *n* (%)				
N0	1271 (87.716)	1211 (89.505)	60 (62.500)	<0.001
N1	178 (12.284)	142 (10.495)	36 (37.500)	
Radiation, *n* (%)				
No	1426 (98.413)	1334 (98.596)	92 (95.833)	0.036
Yes	23 (1.587)	19 (1.404)	4 (4.167)	
Chemotherapy, *n* (%)				
No	277 (19.117)	260 (19.217)	17 (17.708)	0.716
Yes	1172 (80.883)	1093 (80.783)	79 (82.292)	
Surgery, *n* (%)				
No	13 (0.897)	4 (0.296)	9 (9.375)	<0.001
Yes	1436 (99.103)	1349 (99.704)	87 (90.625)	

### 3.3 Correlation analysis

**FIGURE 2:**
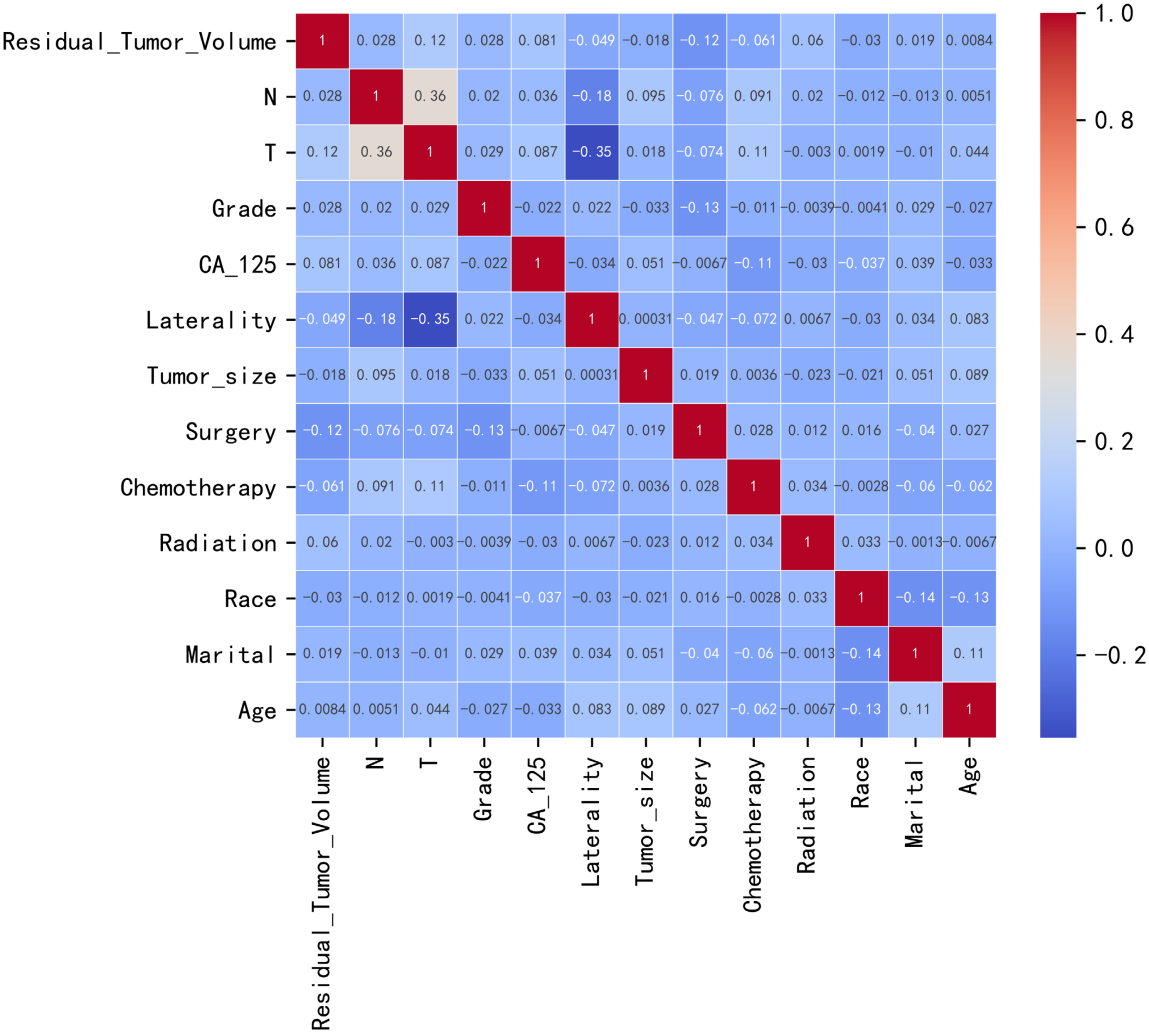
A heatmap representation of the Spearman correlation matrix of the variables. Relevant correlations are color‐coded based on the strength of the correlation.

Spearman correlation analysis was employed to investigate the interrelationships among the variables. The correlation heatmap (Figure 2) illustrated the correlation between each factor, like the correlation coefficient between N and T is 0.36, which is less than 0.5, indicating a weak correlation, and the others are also weak correlations.

### 3.4 Feature variable selection

**FIGURE 3:**
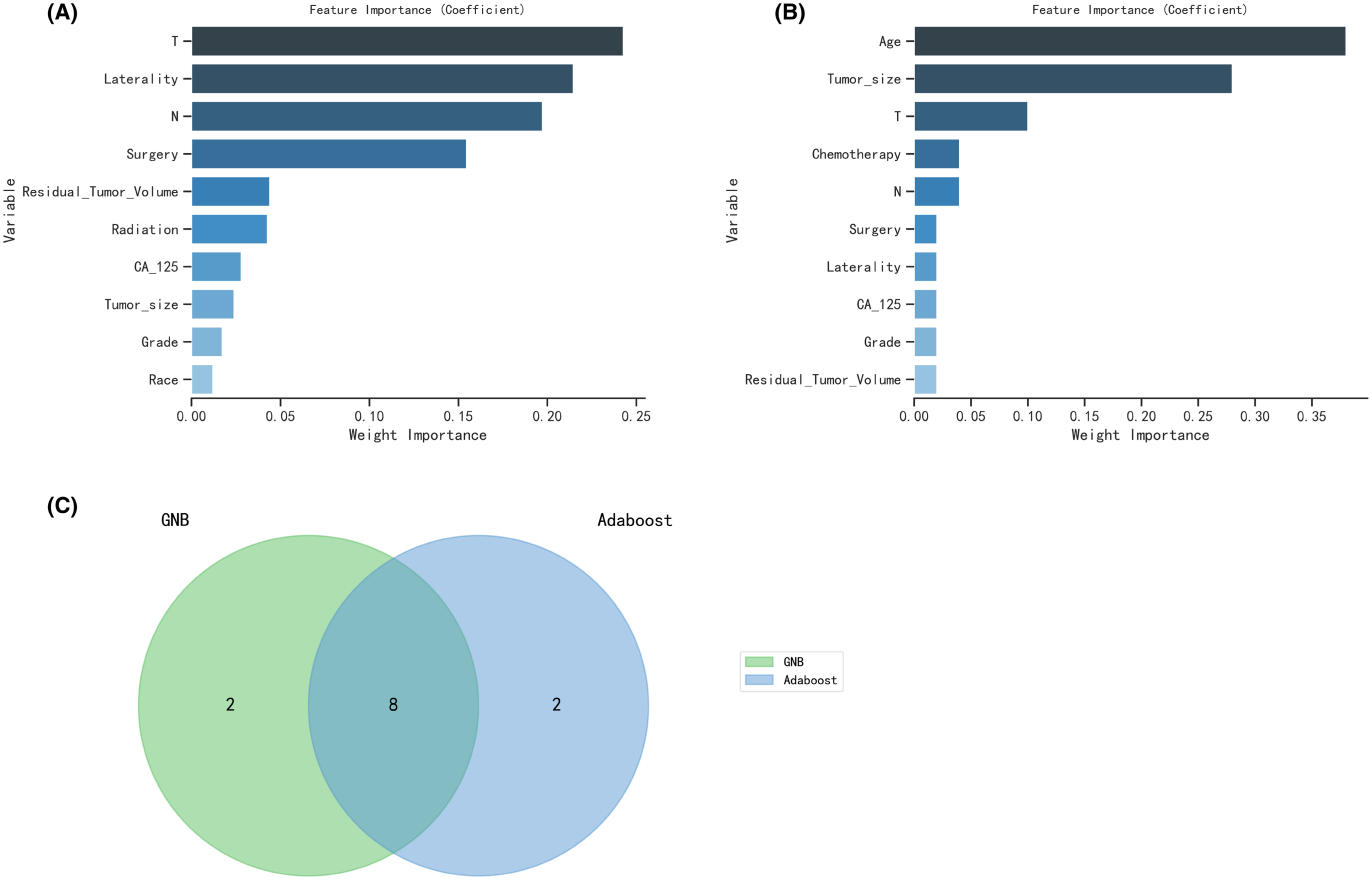
The results of GNB (A) and Adaboost (B) machine learning algorithms filter the top 10 important variables. The results are expressed by coefficient value. (C) Venn analysis of the results of the above two machine algorithms.

The optimization of feature variables was conducted through the application of machine learning algorithms, namely GNB (Figure 3A) and Adaboost (Figure 3B). Each algorithm was employed to identify the top 10 most important feature variables for their respective models. Subsequently, utilizing Venn diagrams, a comprehensive analysis led to the identification of eight variables (Grade, CA125, Surgery, T, Residual Tumor Volume, N, Laterality, and Tumor size) for the construction of the model (Figures 3C).

### 3.5 The predictive performance and calibration of machine learning models

**FIGURE 4:**
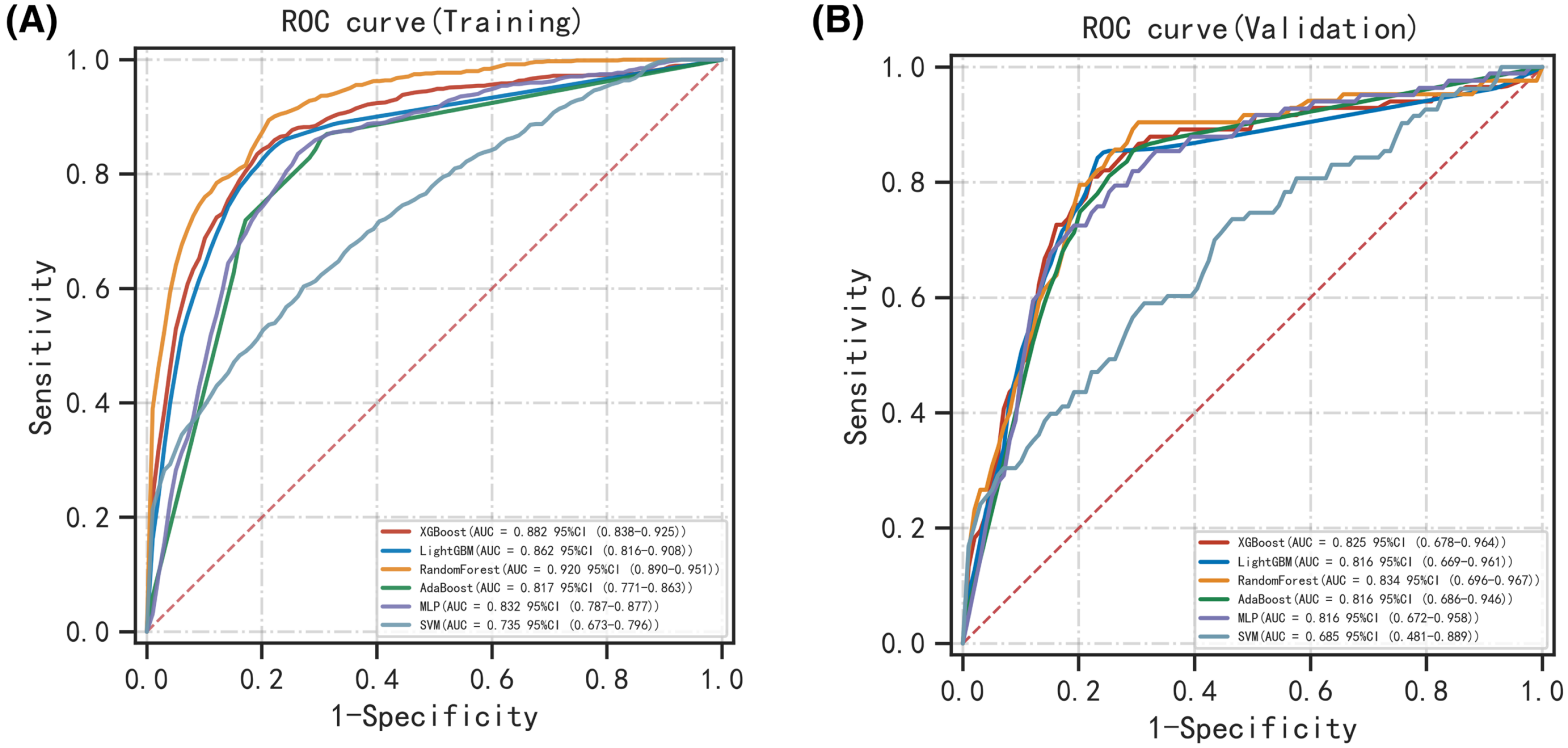
ROC curve comparison of training set (A) and Validation set (B) in multiple machine algorithms.

**FIGURE 5:**
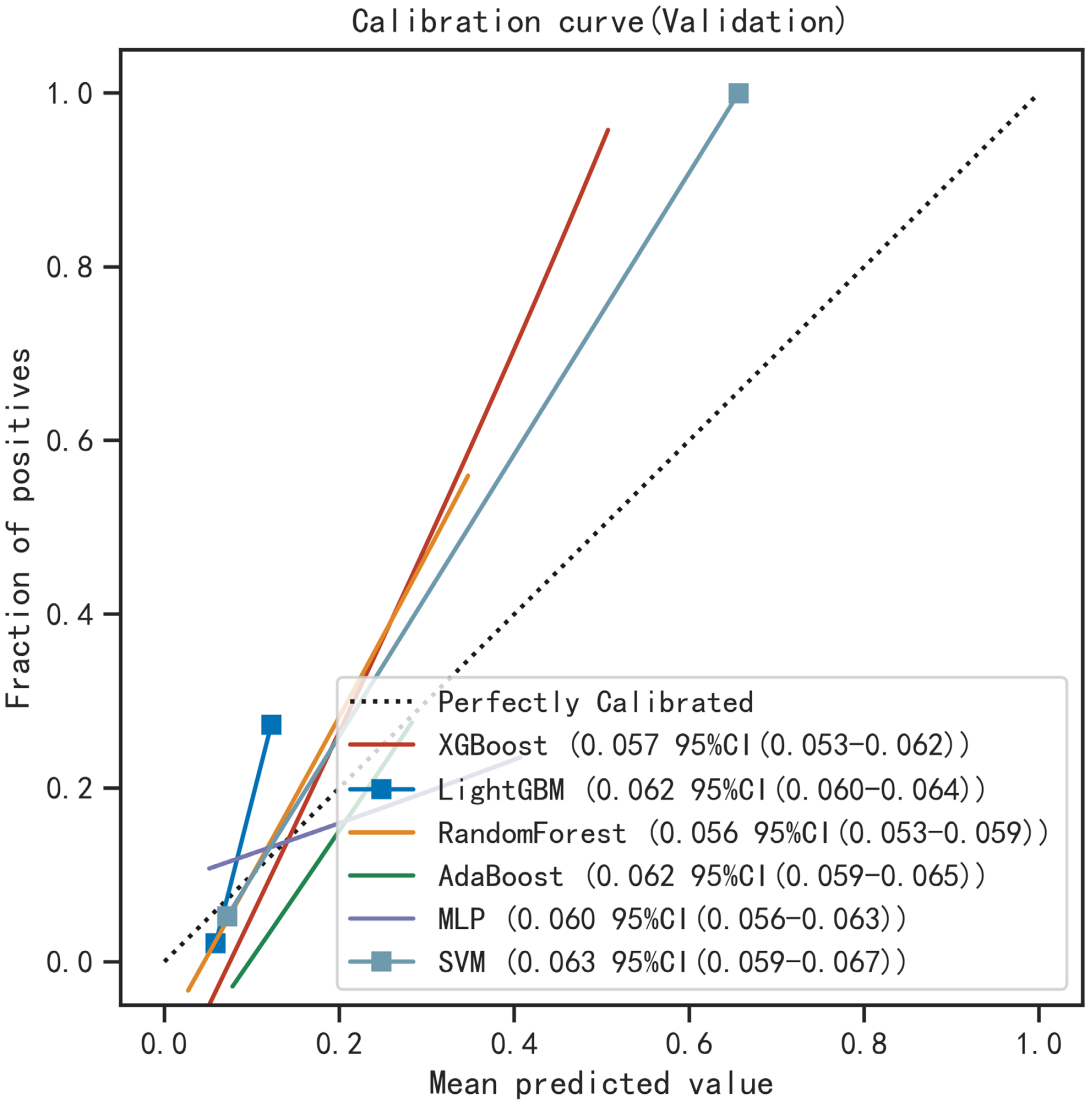
Comparison of multiple machine learning evaluation indicators in the validation set.

In order to establish a predictive model for distant metastasis in ovarian clear cell carcinoma (OCCC) based on machine learning algorithms, we use the eight features (Grade, CA125, Surgery, T, Residual Tumor Volume, N, Laterality, and Tumor size) identified through screening as independent factors. The algorithms employed include XGBoost, LGBM, RF, AdaBoost, SVM, and MLP. To mitigate overfitting and select the optimal model, 10‐fold cross‐validation was performed using the training set, yielding average values for accuracy, sensitivity, specificity, positive predictive value, negative predictive value, F1 score, and AUC for the six machine learning models (Table 3). The results indicate that, for the validation set, the RF model demonstrated superior predictive performance with accuracy, sensitivity, specificity, positive predictive value, negative predictive value, F1 score, and AUC (95% CI) of 0.792 (0.762–0.823), 0.904 (0.835–0.973), 0.759 (0.731–0.787), 0.221 (0.186–0.256), 0.974 (0.967–0.982), 0.353 (0.306–0.399), and 0.834 (0.696–0.967), respectively, surpassing other machine learning models. The performance of each model in the training and validation set is depicted in the Table 3, and the ROC curves for the six models in the training set (Figure 4A) and validation set (Figure 4B) are illustrated. The comparison of multiple machine learning evaluation indicators in the validation set is shown in Figure 5.

**TABLE 3:** Comparison of multiple machine learning evaluation indexes between training set and test set.

Data set	Models	AUC (95% CI)	Accuracy rating (95% CI)	Sensitivity (95% CI)	Specificity (95% CI)	Positive predictive value (95% CI)	Negative predictive value (95% CI)	F1 score (95% CI)
Training set	XGBoost	0.882 (0.838–0.925)	0.819 (0.806–0.831)	0.841 (0.827–0.854)	0.817 (0.803–0.831)	0.259 (0.247–0.271)	0.984 (0.983–0.985)	0.396 (0.383–0.409)
	LightGBM	0.862 (0.816–0.908)	0.813 (0.802–0.824)	0.839 (0.821–0.858)	0.798 (0.783–0.814)	0.250 (0.238–0.262)	0.982 (0.981–0.984)	0.385 (0.372–0.397)
	RandomForest	0.920 (0.890–0.951)	0.818 (0.799–0.837)	0.882 (0.855–0.909)	0.813 (0.791–0.835)	0.268 (0.245–0.290)	0.988 (0.986–0.990)	0.409 (0.387–0.431)
	AdaBoost	0.817 (0.771–0.863)	0.724 (0.701–0.746)	0.854 (0.824–0.884)	0.715 (0.688–0.742)	0.184 (0.171–0.198)	0.982 (0.980–0.985)	0.302 (0.288–0.316)
	MLP	0.832 (0.787–0.877)	0.743 (0.730–0.755)	0.855 (0.839–0.872)	0.735 (0.721–0.749)	0.196 (0.188–0.204)	0.984 (0.982–0.985)	0.319 (0.308–0.329)
	SVM	0.735 (0.673–0.796)	0.750 (0.673–0.827)	0.615 (0.523–0.707)	0.761 (0.672–0.850)	0.200 (0.148–0.252)	0.962 (0.957–0.967)	0.280 (0.238–0.322)
Test set	XGBoost	0.825 (0.678–0.964)	0.806 (0.779–0.833)	0.867 (0.791–0.942)	0.799 (0.756–0.842)	0.238 (0.201–0.275)	0.978 (0.968–0.987)	0.371 (0.321–0.421)
	LightGBM	0.816 (0.669–0.961)	0.806 (0.787–0.825)	0.854 (0.775–0.933)	0.792 (0.773–0.811)	0.229 (0.197–0.261)	0.976 (0.966–0.986)	0.361 (0.314–0.407)
	RandomForest	0.834 (0.696–0.967)	0.792 (0.762–0.823)	0.904 (0.835–0.973)	0.759 (0.731–0.787)	0.221 (0.186–0.256)	0.974 (0.967–0.982)	0.353 (0.306–0.399)
	AdaBoost	0.816 (0.686–0.946)	0.721 (0.686–0.755)	0.833 (0.755–0.912)	0.778 (0.736–0.821)	0.182 (0.163–0.202)	0.981 (0.974–0.988)	0.299 (0.268–0.329)
	MLP	0.816 (0.672–0.958)	0.732 (0.710–0.755)	0.842 (0.760–0.923)	0.777 (0.718–0.836)	0.184 (0.162–0.206)	0.979 (0.972–0.986)	0.301 (0.268–0.334)
	SVM	0.685 (0.481–0.889)	0.743 (0.661–0.825)	0.711 (0.562–0.860)	0.700 (0.595–0.805)	0.182 (0.073–0.291)	0.947 (0.935–0.958)	0.264 (0.158–0.370)

**FIGURE 6:**
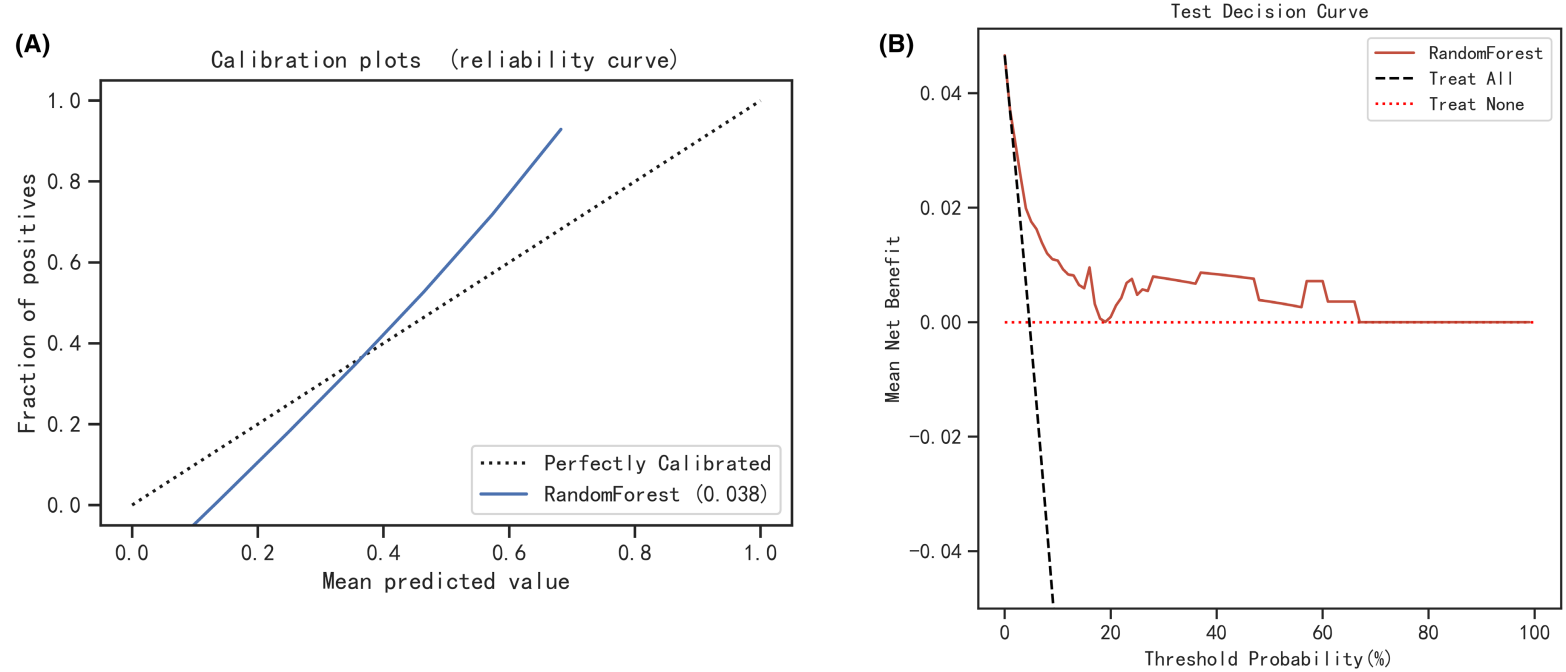
(A) RF model test set calibration curve. (B) RF model test set decision curve analysis (DCA).

**FIGURE 7:**
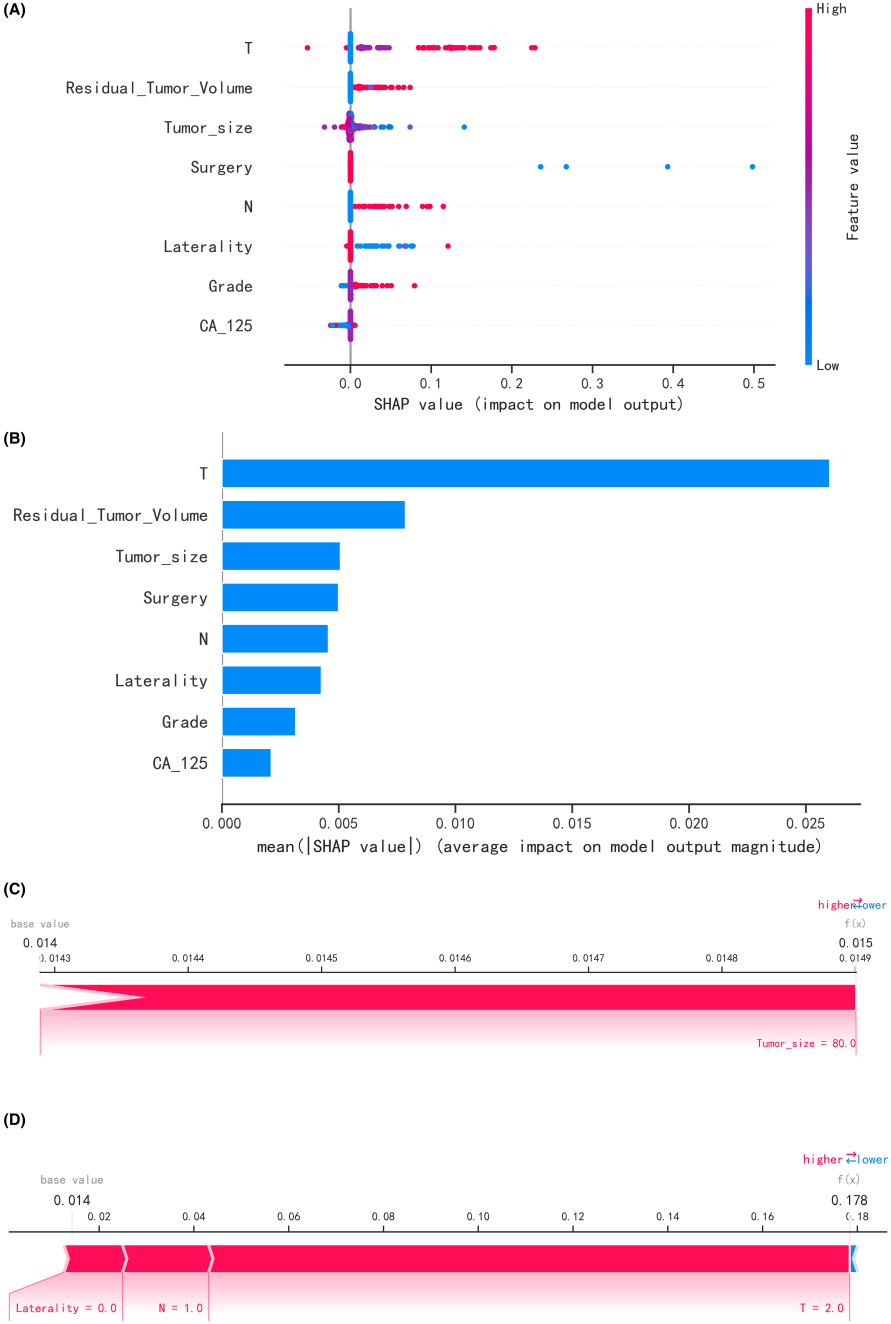
Summary plots of visualize SHAP values. In Figure (A), a point's placement along the x‐axis denotes the actual SHAP value, representing the impact of a specific feature on the model's output for that particular patient. Mathematically, this corresponds to the logarithm of the metastasis risk relative across patients, implying that a higher SHAP value indicates a greater metastasis risk compared to a patient with a lower SHAP value. Features are organized along the y‐axis based on their importance, determined by the mean of their absolute Shapley values. The higher a feature is positioned in the plot, the more significant its impact on the model. Figure B shows the SHAP importance graph of RF algorithm; In Figure C and D, contributing variables are presented in a horizontal line, arranged by the absolute value of their impact. The output value represents the predicted risk of lymph node metastasis, with the base value signifying the expected value of model over the training dataset. These summary plots provide a comprehensive visualization of the explained risk for individual patients, shedding light on the importance and impact of each variable in the context of the model's predictions.

Subsequently, the calibration curve of the RF model was analyzed, demonstrating alignment with the diagonal line, indicative of excellent performance in the test set (Figure 6A), with a Brier Score of 0.038. The decision curve analysis (DCA) curve for the RF model also exhibited favorable net clinical benefit, confirming its excellent performance in the test set (Figure 6B). Utilizing SHAP summary plots (Figure 7A), we computed the contribution of each feature to the model output to identify the most relevant predictive factors. The SHAP importance plot (Figure 7B) further elucidates the impact of individual features on the model.

Based on these findings, we applied the RF model as the final classification model for the test set, with accuracy, sensitivity, specificity, positive predictive value, negative predictive value, F1 score, and AUC (95% CI) of 0.771, 0.692, 0.853, 0.13, 0.981, 0.22, 0.801 (0.655–0.947), respectively (Figure 7C,D).

## 4 DISCUSSION

EOC is a complex and heterogeneous group of diseases characterized by diverse genomic features.^22^ OCCC, a distinct subtype of EOC, poses significant challenges in terms of chemotherapy resistance and poor prognosis.^23^ This research focuses on leveraging machine learning algorithms to analyze clinical data from OCCC patients and construct predictive models for distant metastasis. The study aims to identify risk factors and prognostic indicators, providing valuable insights for enhancing the clinical management and survival prognosis of OCCC patients with distant metastasis.

The prevalence of OCCC varies across racial and geographical groups, with the Asian population, particularly in Japan, exhibiting the highest incidence rates. OCCC was not easily detected in the early stages. Considering the high incidence of lymph node metastasis in this subtype, early OCCC requires extensive staging, including pelvic and para aortic lymph node dissection.

OCCC's resistance to platinum‐based chemotherapy contributes to increased risks of infiltration, metastasis, and relapse, leading to unfavorable prognoses.^24^ This study's emphasis on identifying risk factors for metastasis is crucial, as it addresses the pressing need to improve the survival prospects of OCCC patients.

To identify the most critical risk factors, machine learning algorithms, including GNB and Adaboost, were employed.^25^ The Venn test was used for further refinement. The most important eight risk factors were obtained, including Grade, CA125, Surgery, T, Residual Tumor Volume, N, Laterality, and Tumor size, which suggested that clinical attention should be paid to improving and recording these indicators in patients with OCCC for the assessment of metastasis risk. We further evaluated the predictive performance and calibration of machine learning models, including XGBoost, LGBM, RF, AdaBoost, SVM, and MLP. The Random Forest model demonstrated superior performance, surpassing other models in terms of accuracy, sensitivity, specificity, positive predictive value, negative predictive value, F1 score, and AUC. The thorough evaluation of the model's performance using metrics and visualizations, such as ROC curves, calibration curves, and decision curve analysis, adds credibility to the study's findings. Previous studies have suggested that most EOCs are characterized by peritoneal disseminated metastasis. For advanced EOCs, neoadjuvant chemotherapy (NACT) can be considered to reduce tumor volume, reduce surgical differences, and improve surgical success rates. Recently, new clinical treatment methods have been developed, including immune checkpoint blockade therapy, targeted angiogenesis therapy, the use of ARID1A synthesis for lethal interactions, and targeting liver cell nucleus factor 1 β New therapies such as ferroptosis bring great hope to ovarian cancer patients.^26^ Compared to imaging recognition of distant metastases and staging diagnosis, model provides a new approach. This algorithm is from a statistical perspective, using an RF model and introducing relevant variables to determine the probability of distant metastasis.

The application of the Random Forest model as the final classification model for the test set yielded promising results, emphasizing its potential utility in predicting distant metastasis in OCCC patients. The accuracy, sensitivity, specificity, and other metrics demonstrated the model's ability to provide valuable insights into patient prognosis.

This research contributes to the field of ovarian cancer research by leveraging machine learning techniques to identify and understand the factors influencing distant metastasis in OCCC patients. The predictive models developed in this study have the potential to assist clinicians in making informed decisions regarding the clinical management of OCCC patients, ultimately improving survival outcomes. Future studies could focus on validating these models using external datasets and exploring additional factors as postoperative complications that may contribute to the metastatic behavior of OCCC. Meanwhile, there are perhaps more pressing clinical questions that this could be applied to, such as preoperative risk stratification for malignancy like the O‐RADS system, determining likelihood of a complete gross resection, predicting postoperative complication rates, predicting platinum resistance, etc.

## 5 CONCLUSION

In conclusion, the study successfully leveraged machine learning algorithms, particularly the Random Forest model, to develop a predictive model for distant metastasis in OCCC. The robust performance of the model suggests its potential clinical utility in guiding treatment decisions and improving outcomes for OCCC patients. Further validation and refinement of the model could contribute to its integration into clinical practice for personalized care.

## AUTHOR CONTRIBUTIONS

**Qin‐Hua Guo:** Data curation (equal); formal analysis (equal); methodology (equal); resources (equal); software (equal); validation (equal); visualization (equal); writing – original draft (equal). **Feng‐Chun Xie:** Data curation (equal); formal analysis (equal); methodology (equal); software (equal). **Fang‐Min Zhong:** Funding acquisition (equal); validation (equal); visualization (equal). **Wen Wen:** Validation (equal); visualization (equal). **Xue‐Ru Zhang:** Validation (equal); visualization (equal). **Xia‐Jing Yu:** Validation (equal); visualization (equal). **Xin‐Lu Wang:** Validation (equal); visualization (equal). **Bo Huang:** Funding acquisition (equal); validation (equal); visualization (equal). **Li‐Ping Li:** Conceptualization (equal); project administration (equal); supervision (equal); writing – review and editing (equal). **Xiao‐Zhong Wang:** Conceptualization (equal); funding acquisition (equal); project administration (equal); resources (equal); writing – review and editing (equal).

## FUNDING INFORMATION

The study was funded by the National Natural Science Foundation of China (82160405 and 82160038) and the Natural Science Foundation of Jiangxi Province (20232BAB216037).

## CONFLICT OF INTEREST STATEMENT

No potential conflict of interest was reported by the authors.

## ETHICS STATEMENT

All experimental protocols were approved by the Ethics Committee of The Second Hospital of Nanchang University (Nanchang, China).

## Data Availability

All data used in this work can be acquired from the Surveillance, Epidemiology, and End Results Program (SEER; https://seer.cancer.gov/SEER).
